# A Methodological Approach for Motor Selection in Dental Impression Material Dispensers Using Experimental and Image Analysis Techniques

**DOI:** 10.3390/ma17071467

**Published:** 2024-03-22

**Authors:** Ji-Min Hwang, Sang-Wook Park, Ji-Su Jeong, Ji-Wook Kim, Dae-Cheol Ko, Jin-Seok Jang

**Affiliations:** 1 Advanced Mobility Components Group, Korea Institute of Industrial Technology, Daegu 42994, Republic of Korea; jm0512@kitech.re.kr (J.-M.H.); swpark88@kitech.re.kr (S.-W.P.); jsjeong@kitech.re.kr (J.-S.J.); jwkim0@kitech.re.kr (J.-W.K.); 2 Department of Nanomechatronics Engineering, Pusan National University, Pusan 46241, Republic of Korea

**Keywords:** impression material, dispenser, drive motor, capacity, extrusion load, pressurization speed

## Abstract

This study presents a methodology to prevent the overdesign of electric dispensers for dental impression materials by analyzing the necessary load and determining the appropriate pressurization speed and drive motor capacity. We derived an equation to calculate the required torque and rotational speed of the motor based on the extrusion load and the speed of the impression material. A specialized load measurement system was developed to measure the load necessary to extrude the impression material. Through experiments and image processing, we measured the radius of curvature of the trajectory of the impression material and correlated it with the pressurization speed. Techniques such as position coordinate plotting, curve fitting, and circle fitting were employed to determine the pressurization speed that aligns with the manufacturer’s recommended curvature radius. These findings led to a substantial decrease in the necessary motor torque and rotational speed compared with the current standards. This research provides a systematic approach to sizing drive motors using extrusion load and pressurization speed, aiming to reduce overdesign, power consumption, and the weight and size of the motor and battery, thereby contributing to the development of more efficient and compact dental impression material dispensers.

## 1. Introduction

Dental impression materials are employed to precisely capture the shape and occlusal relationships of teeth and surrounding tissues with the aim of accurate replication [1]. Dispensers mix these materials with hardeners and uniformly deliver them to targeted areas for impressions using impression materials and mixing tips suitable for specific treatment purposes.

Among the array of impression materials, additional silicone types are prevalent in contemporary dental treatments because of their high fidelity in impression capture, rapid setting times, and exceptional mechanical properties [1,2]. These materials are classified into light, medium, and heavy bodies based on their consistency, which refers to the degree of firmness with which particles of a material, when prepared for use, cohere in such a way as to allow the material to flow or resist flow [3]. Light-bodied materials are predominantly used with intraoral tips to ensure detailed flow into the intricate aspects of teeth, whereas medium- and heavy-bodied materials are reserved for tray applications.

The methods for mixing dental impression materials have evolved significantly. Hand mixing with a spatula was once widespread, but the advent of automatic mixing systems has markedly reduced the incorporation of air bubbles, material waste, and time required for mixing [4]. Recently, the prevalence of mechanical mixing systems has increased. These systems use blades within the mixing tip, driven by a motor, to amalgamate the impression material and the hardener. Such systems feature a drive motor that exerts pressure on the piston to extrude the material, coupled with a mixing motor within the tip to ensure thorough blending [5], providing the advantages of complete and rapid mixing with user-friendly operations [6]. Nonetheless, for electric dispensers equipped with mechanical mixing systems, there are no standardized guidelines for the necessary cartridge load or optimal speed for pressurization during extrusion. This absence of standards often results in oversized motors, which unnecessarily increase the weight and size of the dispenser.

Numerous studies have focused on the physical attributes of impression materials, including viscosity, ability to capture fine details, and dimensional stability [7,8,9], in addition to their chemical properties and composition [10]. Significant research has been conducted on the effects of different mixing methods on these materials. Craig et al. established that automatic mixing conserves more than half of the material compared with manual mixing [11]. Keck et al. validated its enhanced efficacy in preventing contamination and in reducing voids [12]. Di Felice et al. reported that mechanical mixing results in fewer voids than manual methods [13], whereas Zelikman et al. highlighted the merits of mechanical mixing for producing more precise dental replicas [14]. Lepe et al. assessed the influence of various mixing techniques on the contact angle, absorption, and mass reduction of impression materials [15]. Nam et al. conducted a survey among dental professionals to evaluate mixing quality, ease of tray loading, and their preferences relating to mixing techniques [16]. Furthermore, Maluly-Proni et al. investigated the effect of the shape of mixing tips on the dimensional stability, fidelity of fine detail replication, and material waste post-mixing [17]. From a rheological perspective, German et al. investigated the viscoelastic properties of impression materials using a dynamic stress rheometer [18,19] and Martinez et al. explored their thixotropic behavior [20]. These investigations have contributed to the advancement of impression material quality but have fallen short of guiding the mechanical design aspects of dispensers, such as the optimal load to be applied to the cartridge and the appropriate speed for material extrusion.

This study formulated a methodology for determining the necessary load for extruding dental impression materials and the optimal pressurization speed with the aim of mitigating overdesign in electric dispensers. Through an examination of the power transmission mechanism of the electric dispenser, formulas were deduced to ascertain the requisite torque and rotational speed of the drive motor, factoring in the extrusion load, and pressurization speed. A load measurement system was engineered to gauge the extrusion load of the impression materials across the various experimental setups. Moreover, an approach was developed to calculate the suitable pressurization speed for the load exerted on the cartridge by utilizing image analysis of the extrusion trajectory. Integrating the data on impression material extrusion load with trajectory image analysis into the formulated equations enabled the development of a technique for estimating the optimal capacity of the drive motor, which is adaptive to the attachment of an intraoral tip.

## 2. Power Transmission Analysis

### 2.1. Structure of an Electric Dispenser

**Figure 1:**
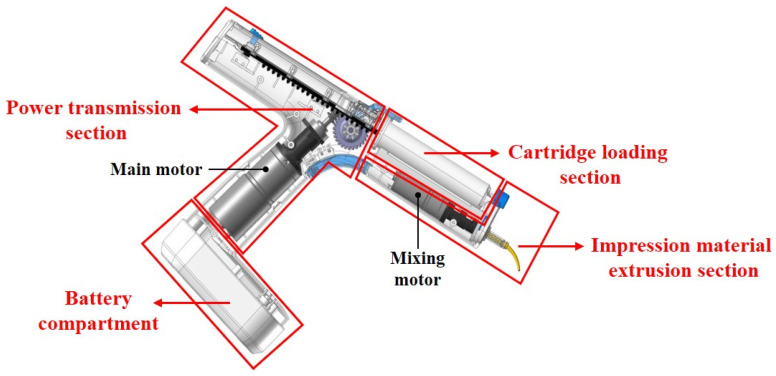
Structure of dental impression material electric dispenser.

Figure 1 shows a schematic of the electric dispenser used for dental impression materials. The dispenser examined in this study (Dongheungplatech, Daegu, Republic of Korea) is of the electric gun type, comprising a primary battery compartment, power transmission section, cartridge loading section, and extrusion section for the impression material. The power transmission section is outfitted with a main motor that initiates rotation when energized by the battery cells and is connected to a gear assembly. The extrusion section was engineered to link the cartridge outlet to the mixing tip, thereby facilitating the combination and external extrusion of the impression material and hardener through the mixing motor.

The brief dimensions of the electric dispenser are as follows: the maximum height from the battery compartment to the top is 237.05 mm, the maximum length from the rear where the rack is located to the extrusion section is 272.32 mm, and while the width of the dispenser is almost uniform throughout, it reaches up to 49.96 mm.

### 2.2. Power Transmission Mechanism

**Figure 2:**
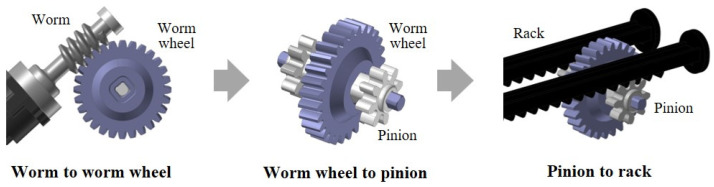
Power transmission mechanism.

Figure 2 presents the sequence of the power transmission mechanism within the electric dispenser. This section houses the main motor coupled with a gear assembly that includes a worm, worm wheel, pinion, and rack.

The operational sequence of the dispenser’s power transmission is as follows. The main motor initially relays power to the worm, which shares its axis. This worm subsequently imparts rotational force to the meshed worm wheel, featuring a gear ratio of 1:28 with the worm. The pinion, affixed to the axis of the worm wheel, rotates concurrently and conveys power to the engaged rack, thereby transforming the rotational force into linear motion. This rack progresses linearly towards the mounted cartridge, exerting pressure on the piston located at the rear end of the cartridge. The ensuing pressure is transmitted to the impression material within the cartridge, compelling extrusion of the material through the outlet.

### 2.3. Equations for Calculating Required Motor Torque and Rotational Speed

Formulas were derived from the power transmission mechanism to calculate the required torque and rotational speed (RPM) of the main motor, based on the extrusion load of the impression material and pressurization speed. The formula to determine the main motor’s necessary torque using the impression material’s extrusion load is as follows:

(1) $$T_{pinion,\;rack}=r_{pinion}\times F_{rack,\;cartridge}$$

In Equation (1), $T_{pinion,\;rack}$ is the torque transmitted by the pinion to the rack, measured in kgf·cm. $r_{pinion}$ is the radius of the pinion, which is 0.6 cm in this study. $F_{rack,\;cartridge}$ is the force applied perpendicularly by the rack to the piston in the cartridge containing the impression material and hardener, representing the extrusion load of the impression material.

The worm wheel, connected to the same axis as the pinion, rotates at the same speed as the pinion. The formula for calculating the required torque of the worm, which transmits power to the worm wheel, is presented as follows:

(2) $$T_{worm,\;wheel}=T_{pinion,\;rack}\times \frac{1}{i_{worm,\;wheel}}\;$$

In Equation (2), $T_{worm,\;wheel}$ is the torque transmitted by the worm to the worm wheel. *i_worm,wheel_* is the gear ratio between the worm and worm wheel, which is 28 in this study. Finally, considering the safety factor, the formula to calculate the required torque of the final motor is presented as follows:

(3) $$T_{motor}=SF\times T_{worm,\;wheel}$$

In Equation (3), $T_{motor}$ and SF are the required torque of the main motor and the safety factor, respectively. Therefore, the process above can be expressed as a single formula, as shown in the following Equation (4).

(4) $$T_{motor}=SF\times \frac{r_{pinion}\times F_{rack,\;cartridge}}{i_{worm,\;wheel}}$$

Next, the formula for calculating the appropriate RPM of the main motor using the appropriate pressurization speed is presented as follows:

(5) $$\omega_{pinion}=v_{rack}\times \frac{60}{2\pi }\times \frac{1}{r_{pinion}}$$

In Equation (5), $\omega_{pinion}$ is the angular velocity of the pinion, that is, its rotational speed, with the unit in RPM. $v_{rack}$ is the translational speed of the rack, which indicates the appropriate pressing speed of the cartridge. Subsequently, the calculation was performed as follows, using the gear ratio of the worm and worm wheel.

(6) $$\omega_{worm}=\omega_{worm}\times i_{worm,\;wheel}$$

In Equation (6), $\omega_{worm}$ is the rotational speed of the worm. Because the worm and main motor are connected on the same axis, the rotational speed of the worm indicates the appropriate rotational speed of the main motor. This process is also expressed as a single equation, as shown in Equation (7), where $\omega_{motor}$ is the rotational speed of the main motor.

(7) $$\omega_{motor}=\frac{30\times v_{rack}\times i_{worm,\;wheel}}{\pi \times r_{pinion}}$$

## 3. Measurement of Impression Material Extrusion Load

### 3.1. Load Measurement System

**Figure 3:**
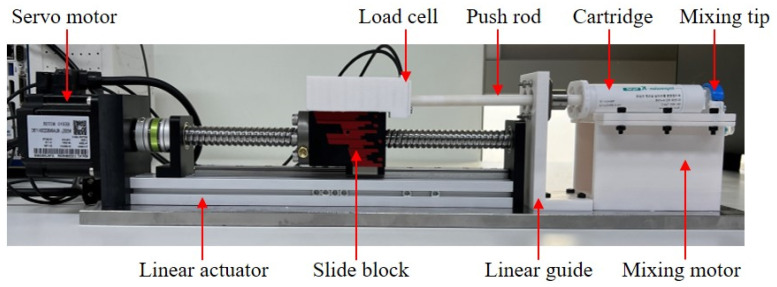
Structure of load measurement system testbed.

A system was devised to measure the load needed to be applied to the piston in the cartridge when extruding impression material through the mixing tip. Figure 3 displays the testbed’s structure that constitutes the load measurement system. The overall dimensions of the testbed are as follows: the maximum height from the bottom to the top is 110 mm, the maximum length from the servo motor to the mixing tip is 579.5 mm, and the maximum width is 100 mm. The components and specifications of the testbed are detailed in Table 1. The testbed employs a linear actuator to simulate a power transmission mechanism that directs the rack towards the cartridge. It includes a servo motor, ball screw, load cell mount, pushrod, linear guide, and section for cartridge loading, as depicted in Figure 3. The speed of the servo motor is regulated by the LabVIEW 2020 program. The rotational movement of the servo motor is transformed into linear motion via the ball screw. A load cell mount, along with the load cell, was affixed to the sliding block. The load cell gauges the force applied to the cartridge through the pushrod, ensuring that the cartridge remains fixed and resistant to displacement under the applied load. Additionally, the data from the load cell are logged by the load measurement program, specially developed using LabVIEW. A linear guide fitted with ball bearings was installed at the end of the linear actuator to facilitate smooth linear motion of the pushrod. At the forefront of the pushrod guide, a mixing motor, mixing tip, and cartridge mounting section are integrated to emulate the dispenser’s extrusion section.

### 3.2. Load Measurement Experiment

**Figure 4:**
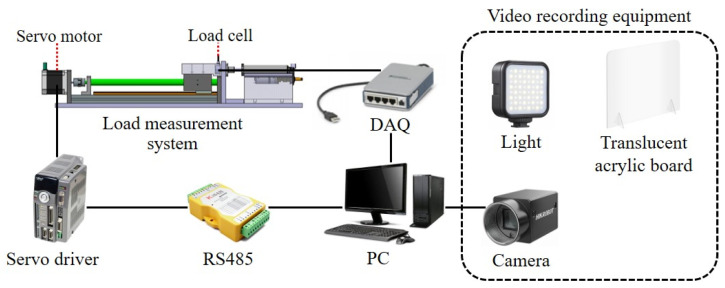
Schematic diagram of experimental setup.

A load measurement system was developed to assess the load necessary for extrusion of the impression material through the mixing tip. Figure 4 illustrates the schematic of the experimental setup. The load cell data were acquired using DAQ equipment (National Instruments, Austin, TX, USA), and the RS485 communication protocol device facilitated communication between the PC program and servo motor. Additionally, the speed of the servo motor was controlled using a specialized LabVIEW-based servo motor control program.

Table 2 illustrates the characteristics and physical properties of elastomeric impression materials in accordance with the international standard (ISO 4823) [3] for dental impression materials. Impression materials are classified into types 0, 1, 2, or 3 based on the established consistencies determined immediately after completion of mixing, corresponding, respectively, to putty, heavy-bodied, medium-bodied, and light-bodied consistencies [3]. The consistency of impression materials is associated with viscosity, indicating that a lower consistency corresponds to higher viscosity, while a higher consistency implies lower viscosity [1,21]. The impression materials utilized in our experiments are types 2 and 3. Within the constrained types specified by ISO standards, the differences in the properties of the impression materials are minimal. Therefore, this approach can be uniformly applied regardless of variations in the properties of the impression materials used, guaranteeing reliable results.

In this study, various domestically produced addition silicone impression materials with different consistencies, namely, light body and medium body, were employed. The comprehensive details of the dental impression materials used are presented in Table 3.

**Figure 5:**
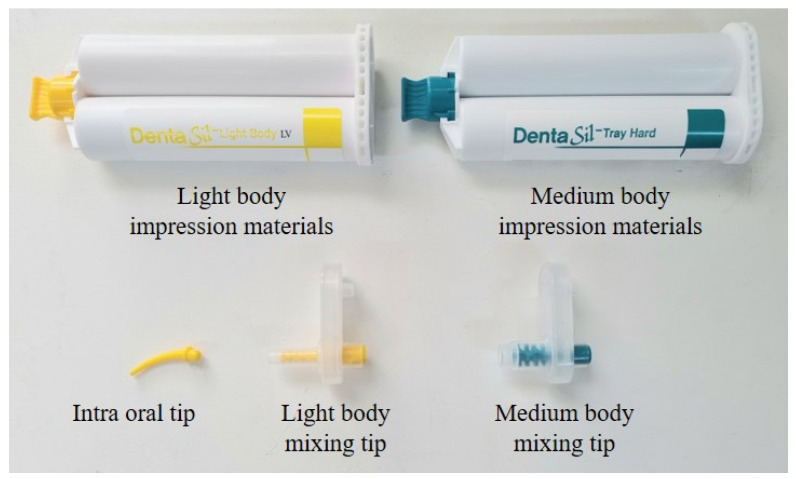
Experimental equipment and materials.

Mixing tips were used based on the variety of impression materials used. The experiment also incorporated the use of an intraoral tip for precise injection into teeth. Figure 5 shows the impression materials, mixing tips, and intraoral tips utilized in the experiment.

The controlled variables in the load measurement experiment included the type of impression material, mixing tip, and the operation of the mixing motor. The decision to exclude the intraoral tip from the experiment was based on the following considerations. Conventional electric dispensers typically operate at a pressurization speed of 1 mm/s and are optimized for use without an intraoral tip. In contrast, when utilizing a light body impression material, which is generally applied with an intraoral tip, the extrusion outlet area is significantly reduced.

**Figure 6:**
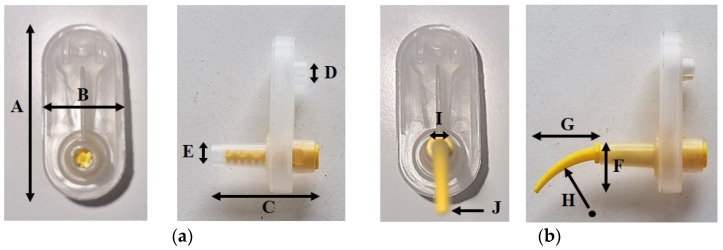
Before and after intraoral tip attachment: (**a**) Before; (**b**) After.

Figure 6 and Table 4, respectively, illustrate the appearance and dimensions of the light body mixing tip before and after the attachment of the intraoral tip. This indicates that, with the attachment of the intraoral tip, the impression material is extruded through an additional longer and narrower passage. Table 5 presents the change in exit area before and after attaching the intraoral tip, calculated based on the measured dimensions. Upon attaching the intraoral tip, it was observed that the extrusion outlet area is reduced to merely 5% of the original mixing tip’s outlet area when using a light body impression material.

Applying the same pressurization speed to a significantly reduced outlet area generates excessive pressure on the components of the extrusion section. Although the impression material flows into the mixing tip at a consistent rate due to the load applied by the rack, the reduced outlet area results in excessive pressure within the mixing tip. This can lead to leakage of the impression material and hardener and, in severe cases, damage to the plastic injection-molded mixing tip. Consequently, using light body impression material with an intraoral tip was identified as the most extreme condition for the dispenser.

The pressurization speed of the pushrod was set to 1 mm/s, mirroring the progress speed of the standard dispenser rack, ensuring full pressurization of the cartridge. Additionally, acknowledging the impact of temperature on the properties of impression material [22,23], all experiments were performed at a controlled room temperature of 20 °C. The detailed experimental conditions are outlined in Table 6. Each experiment was conducted twice to ensure data reproducibility and reliability.

## 4. Analysis of Appropriate Pressurization Speed

### 4.1. Criteria for Selecting Appropriate Pressurization Speed

Maintaining the conventional dispenser pressurization speed of 1 mm/s while using a light body impression material with an intraoral tip may result in excessive conditions, potentially leading to various issues, as previously discussed. Therefore, experiments were conducted to identify the optimal pressurization speed under such extreme conditions.

Two criteria were established to determine the appropriate pressurization speed for the experiment. First, the speed was set to prevent leakage and damage to components. Second, the speed was chosen to be similar to the recommended curvature radius of the impression material trajectory provided by the manufacturer of the electric dispenser and dental equipment used in this study, which is the curvature radius of the intraoral tip (30.33 mm).

### 4.2. Appropriate Pressurization Speed Analysis Experiment

**Figure 7:**
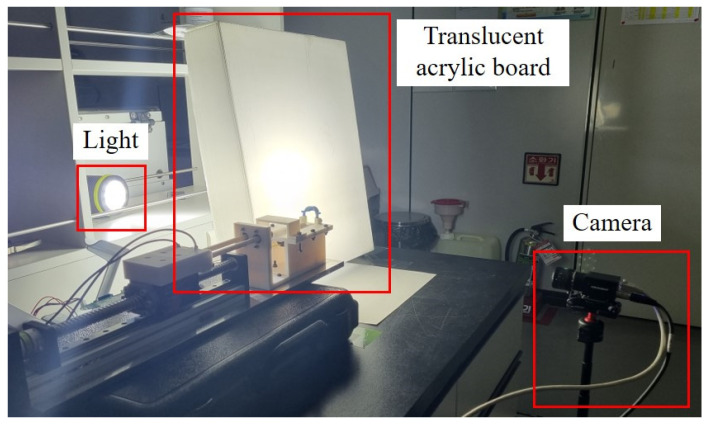
Video recording equipment.

Utilizing the experimental equipment depicted in Figure 4 and video recording devices, we conducted experiments to analyze the trajectory of the impression material extruded at various pressurization speeds. The rotational speed of the servomotor was modulated using the control program to create different pressurization conditions. To capture the trajectory clearly, a Hikrobot camera was positioned along the outlet of the intraoral tip. Adequate lighting and a translucent acrylic panel were employed to ensure that the recordings were clear and shadow-free. Figure 7 displays the actual video recording setup.

To determine the optimal pressurization speed for the light body impression material with an intraoral tip, we established the experimental conditions, as detailed in Table 7. The range of pressurization speeds in the experiment was set from 0.3 to 0.8 mm/s, based on preliminary tests. At speeds below 0.2 mm/s, the impression material extruded too slowly and fell directly to the ground because of gravity immediately after exiting the outlet, making it impractical for precise injections into teeth and general application. Conversely, speeds above 0.9 mm/s resulted in leakage at the mixing tip and component junctions, as well as damage to the mixing tip, leading to their exclusion from the experiment. Furthermore, to analyze the trajectory of the mixed impression material and hardener, all the experiments were conducted using a mixing motor during operation.

Following the application of the determined pressurization speed for each condition, the cartridge was pressurized for 80 s, and the trajectory of the extruded impression material through the intraoral tip was recorded. The recordings were recorded at 15 frames per second (fps). Each experiment was performed three times to enhance the reproducibility and reliability of the data.

### 4.3. Image Processing

The footage capturing the extrusion trajectory of the impression material was then subjected to image processing. Initially, segments depicting stabilized extrusion behavior and load were selected. A MATLAB program was developed for the image processing. First, the RGB values in the trajectory area were extracted. Specific color coordinates from the image were isolated using code to map the position coordinates of the extrusion trajectory of the impression material. The actual vertical length of the intraoral tip, positioned on the same plane as the trajectory, was compared with the corresponding number of pixels in the image to accurately reflect the actual length in the graph and the results.

Furthermore, curve fitting was applied to consolidate the numerous points of the trajectory coordinates into a single line [24]. Curve fitting was performed utilizing both polynomial regression and exponential model regression utilizing the following equations [25]. In the case where the degree of the polynomial is m−1, it can be represented by the following function. In this study, x and y, respectively, denote the positional coordinates of pixels extracted along the trajectory of the impression material extrusion.

(8) $$f\left(x\right)=\sum_{j=1}^{m}a_{j}x^{j-1},\;\;\;\;\;\;j=1,\;2,\;\cdots ,m$$

The basis functions for the aforementioned equation are as follows:

(9) $$f_{j}\left(x\right)=x^{j-1},\;j=1,\;2,\;\cdots ,m$$

The function is to be fitted to n data points $\left(x_{i},\;y_{i}\right),\;i=1,\;2,\;\cdots ,\;n$. To mitigate the noise present in the data, a least-squares fit was utilized, minimizing the expression with respect to $a_{j}$, as outlined in Equation (10).

(10) $$S(a_{1},\;a_{2},\;\cdots ,a_{m})={\sum_{i=1}^{n}\left[y_{i}-\sum_{j=1}^{m}a_{j}f_{j}\left(x_{i}\right)\right]}^{\;2}$$

Therefore, the optimal values of the parameters are given by the solution of the equations.

(11) $$\frac{\partial S}{\partial a_{k}}=-2\left\{\sum_{i=1}^{n}\left[y_{i}-\sum_{j=1}^{m}a_{j}f_{j}\left(x_{i}\right)\right]f_{k}\left(x_{i}\right)\right\}=0,\;\;\;\;\;k=1,\;2,\;\cdots ,\;m$$

In matrix notation these equations are

(12) Aa=b

A and b can be derived as follows:

(13) $$A_{kj}=\sum_{i=1}^{n}{x_{i}}^{j+k-2}\;\;\;\;\;\;\;\;\;b_{k}=\sum_{i=1}^{n}{x_{i}}^{k-1}y_{i}$$

Ultimately, the results enable the calculation of the parameter a value.

Fitting with an exponential function was conducted, employing the exponential model as outlined in Equation (14).

(14) $$f\left(x\right)=ae^{bx}$$

In Equation (14), a transformation to linear regression was achieved by applying ln y instead of y. The function is to be fitted to the data points ($x_{i},\;{\ln y}_{i}$), i=1, 2, ⋯, n.

(15) $$F\left(x\right)=\ln f\left(x\right)=\ln a+bx\;$$

The residuals of the logarithmic fit can be represented as follows:

(16) $$R_{i}=\ln y_{i}-\ln a-bx_{i}=\ln\left(1-\frac{r_{i}}{y_{i}}\right)$$

This discrepancy can be largely eliminated by weighting the logarithmic fit. If the original data residuals *ri* are sufficiently small (*ri* ≪ *yi*), employing the approximation $ln(1-r_{i}/y_{i})\approx r_{i}/y_{i}$ allows for the following assumption:

(17) $$R_{i}\approx r_{i}/y_{i}$$

By minimizing $\sum {R_{i}}^{2}$, weights of $1/\;y_{i}$ can be introduced. This effect can be negated by applying weights of $y_{i}$ when fitting $F\left(x\right)$ to ($lny_{i},x_{i}$). That is, it is minimized as per the following Equation (18).

(18) $$S(a,b)=\sum_{i=1}^{n}{y_{i}}^{2}{R_{i}}^{2}$$

Subsequently, a process identical to that described in Equations (11)–(13) was applied, involving the differentiation of S with respect to the constituting parameters, ultimately yielding the values of a and b.

Thereafter, the curvature radius of the curve-fitted line was determined using circle fit code.

## 5. Results

### 5.1. Impression Material Extrusion Load Results

**Figure 8:**
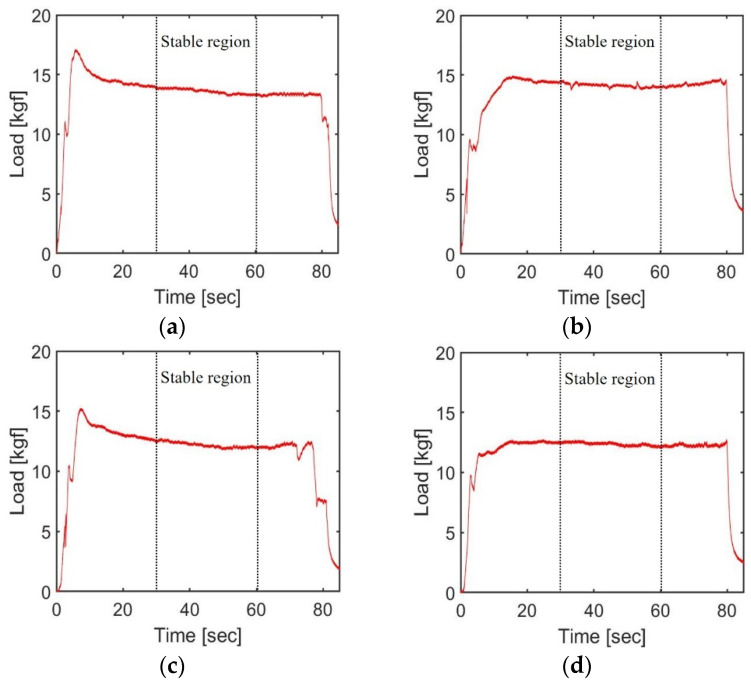
Results of extrusion load using light body impression materials: (**a**) Mixing motor off, 1st test; (**b**) Mixing motor off, 2nd test; (**c**) Mixing motor on, 2nd test; (**d**) Mixing motor on, 2nd test.

**Figure 9:**
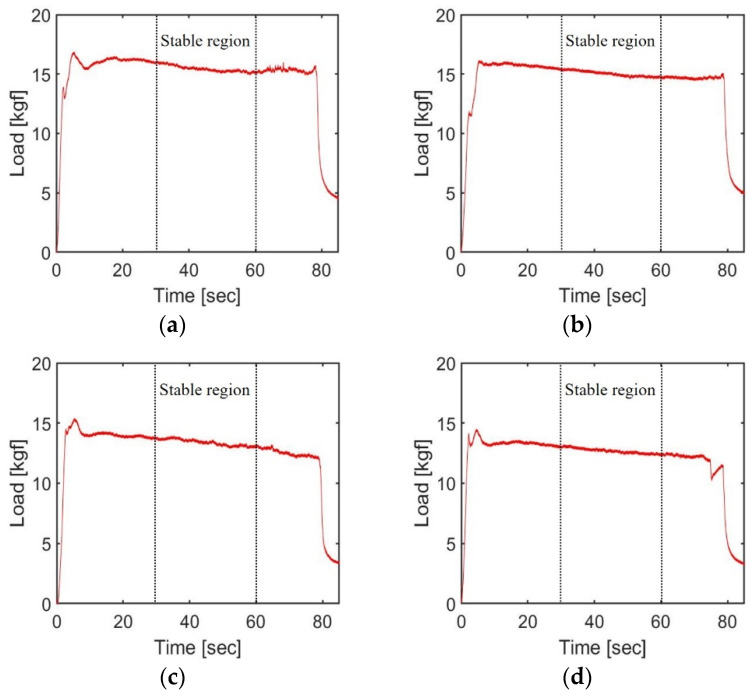
Results of extrusion load using medium body impression materials: (**a**) Mixing motor off, 1st test; (**b**) Mixing motor off, 2nd test; (**c**) Mixing motor on, 2nd test; (**d**) Mixing motor on, 2nd test.

Figure 8 and Figure 9 present the loads measured by the load cell under the various experimental conditions for the light body and medium body impression materials, respectively. Table 8 displays the maximum extrusion load and average of the steady-state load during the 30 to 60 s interval for each experimental setup. These values were derived from the mean of two repeated experiments. The maximum extrusion load was utilized as a key parameter in calculating the torque required for motor design and ensuring a safe design. However, because the maximum extrusion load can be significantly affected by the initial alignment of the plastic cartridge piston, the average load during the stable period was also considered for a comprehensive comparison of results across different experimental conditions.

The highest load in all the experimental cases was recorded when using the medium body impression material without the operation of the mixing motor, reaching a maximum extrusion load of 16.52 kgf. Moreover, under similar conditions, using the medium body impression material generally resulted in a 3–7% higher load compared to using the light body impression material, which is likely due to the higher density of the medium body material. The density of the medium body impression material is approximately 12% higher than that of the light body material, as indicated in Table 8. Andrzej Banaszek et al. have discovered that the load on the drive system increases when pumping liquids with a higher density [26,27]. When referencing this research, it appears that, in the context of applying force to the piston for the extrusion of impression materials, the difference in density has a more significant impact on the measured load than the differences in consistency and viscosity.

The operation of the mixing motor also significantly influenced the extrusion load. In both the medium and light body cases, the extrusion load was 14.48% and 10.58% higher, respectively, when the mixing motor was inactive. This indicates that the extrusion load is relatively low when the impression material and hardener are mixed. This reduction in load when the mixing motor is operated is attributed to the shear-thinning of the impression material, where its viscosity decreases under the shear stress applied during mixing [2,28].

### 5.2. Appropriate Pressurization Speed Analysis

**Figure 10:**
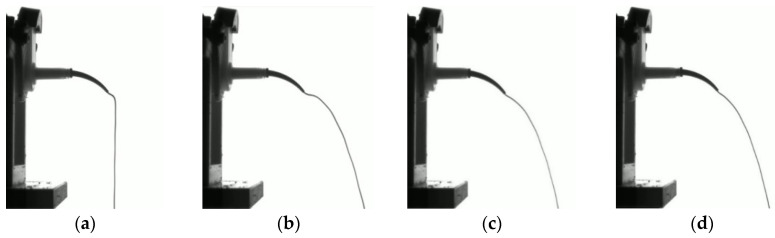
Impression material extrusion behavior over time (pressurization speed: 0.5 mm/s): (**a**) After 10 s; (**b**) After 30 s; (**c**) After 50 s; (**d**) After 70 s.

The optimal pressurization speed according to the established criteria was determined through experimental analysis and image processing. Initially, we assessed the extrusion behavior over time to capture stable segments of the extrusion trajectory from the video recordings. Figure 10 illustrates the time-lapse images of the extrusion behavior of the impression material.

At 10 s after the onset of extrusion, the impression material did not sufficiently reach the mixing and intraoral tips, leading to a noticeably low extrusion volume. After 30 s, the extrusion increased, but it exhibited an unstable behavior and wavy flow.

**Figure 11:**
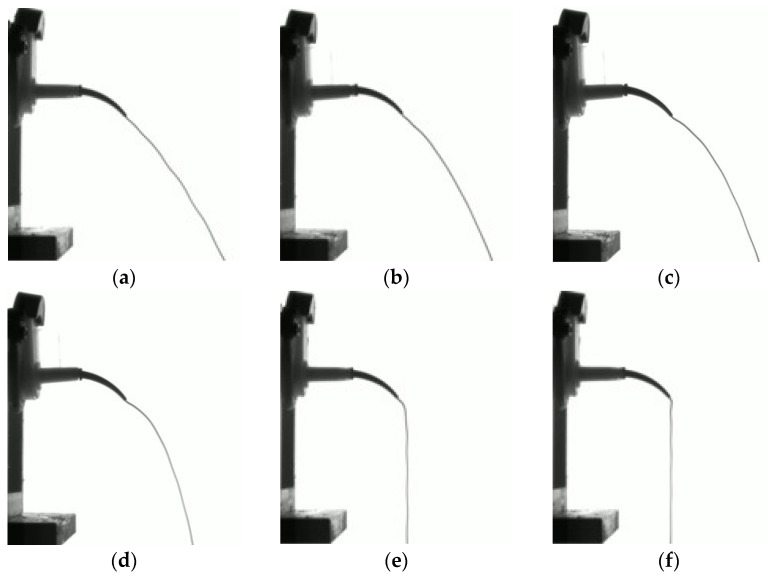
Impression material extrusion trajectory depending on pressurization speed: (**a**) 0.8 mm/s; (**b**) 0.7 mm/s; (**c**) 0.6 mm/s; (**d**) 0.5 mm/s; (**e**) 0.4 mm/s; (**f**) 0.3 mm/s.

At the 50 s mark, the flow line stabilized, exhibiting a consistent form without significant waviness. Consequently, the trajectory 50 s after the start of extrusion was selected for analysis under all pressurization speed conditions, as shown in Figure 11.

The trajectory of the extruded material tended to be straighter at higher pressurization speeds and extended further at the same vertical height. In contrast, at lower pressurization speeds, the trajectory displayed a sharper curve with a smaller curvature radius. At a pressurization speed of 0.3 mm/s, the impression material was observed to fall vertically to the ground immediately after exiting the intraoral tip, which was influenced by gravity.

**Figure 12:**
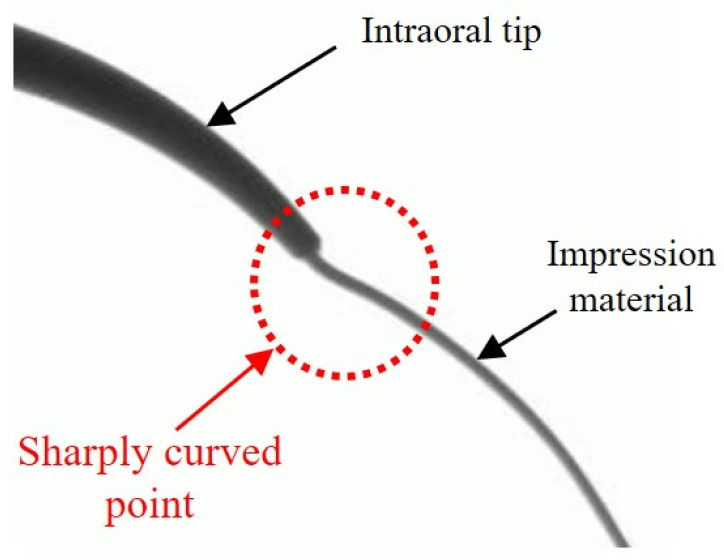
Bending of impression material after intraoral tip.

Furthermore, as depicted in Figure 12, an initial upward curvature in the extrusion trajectory of the impression material was observed immediately after exiting the intraoral tip. This phenomenon is attributed to the velocity distribution differences in the impression material within the curved intraoral tip, leading to pressure variations caused by the centrifugal force [29,30]. This effect was more noticeable at lower pressurization speeds. This is because higher fluid velocities in a curved tube increase the Reynolds number, which, in turn, allows the fluid to escape the effects of centrifugal forces caused by curvature, resulting in a flow pattern similar to that in a straight tube [30].

Impression materials display shear-thinning behavior, where viscosity diminishes and flowability enhances under shear stress [2,30]. Consequently, as the speed of the impression material increases, particularly during mixing and extrusion through the narrow intraoral tip, greater shear stress is applied, leading to a reduction in viscosity. This effect contributes to a more significant increase in the Reynolds number, further accentuating this phenomenon.

(19) $$Re=\frac{\rho VL}{\mu }$$

The Reynolds number is defined as in Equation (19). To elucidate further, in this study, ρ represents the density of the impression material, V is the flow velocity of the material passing through the intraoral tip, L is the diameter of the intraoral tip, and μ is the viscosity of the impression material. Across all the experimental conditions, while the density of the impression material and the diameter of the intraoral tip remain constant, an increase in pressurization speed leads to an increase in the flow velocity V. This increase in velocity results in elevated shear stress, inducing shear-thinning behavior that decreases the viscosity μ of the impression material, consequently leading to an increase in the Reynolds number.

**Figure 13:**
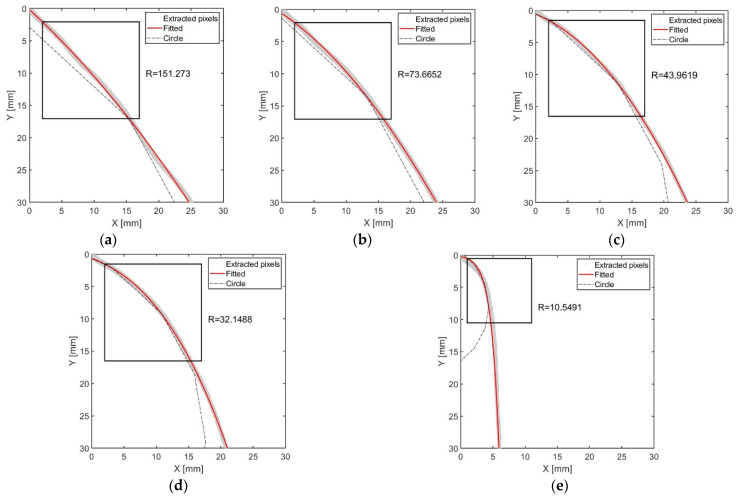
Graphs of extruded the impression material trajectory using image processing (2nd test): (**a**) 0.8 mm/s; (**b**) 0.7 mm/s; (**c**) 0.6 mm/s; (**d**) 0.5 mm/s; (**e**) 0.4 mm/s.

Figure 13 illustrates a graph of the extrusion trajectory, processed from the video footage. The *x*-axis indicates the distance in the direction of the impression material extrusion, while the *y*-axis denotes the distance from the outlet of the intraoral tip in the gravitational direction. The pixel positions extracted from the trajectory and curve-fitted line are represented by grey dots and a red line, respectively.

For the extrusion speeds of 0.8, 0.7, 0.6, and 0.5 mm/s, polynomial fitting was executed as described in Equations (8)–(13). Utilizing the derived parameters, linear regression was performed using a cubic polynomial, and the fitting curve for the extrusion trajectory at extrusion speeds exceeding 0.5 mm/s was plotted on the graph. However, at an extrusion speed of 0.4 mm/s, the trajectory exhibited a form with a sharply high curvature, indicating limitations when using polynomial regression analysis. Consequently, fitting with an exponential function was conducted, employing the exponential model as outlined in Equations (14)–(18). Through this methodology, the exponential function fitting curve of the extrusion trajectory for an extrusion speed of 0.4 mm/s was plotted on the graph.

Using the circle fit code, the average curvature radius within the measured area of the impression material’s extrusion trajectory was calculated. The measurement area was defined as a 15 mm square starting 2 mm from the outlet of the intraoral tip in the extrusion direction. The 2 mm gap was chosen to eliminate the influence of the initial curvature post-extrusion. This square measurement area facilitated a comparison with the manufacturer-recommended curvature radius and excluded the section where gravity caused the material to fall vertically.

Regions where the curvature radius approached infinity, which is indicative of vertical falling due to gravity, were omitted from the measurement. Hence, for a pressurization speed of 0.4 mm/s, the position and dimensions of the measurement area were adjusted as depicted in Figure 13e, and the 0.3 mm/s condition was excluded from the analysis.

Table 9 lists the average curvature radius within the square measurement area, categorized by pressurization speed. The average curvature radius was found to be larger at higher pressure speeds, with a corresponding increase in magnitude. This also correlates with the shear-thinning behavior of the impression material, where viscosity decreases as it passes through the narrow diameter intraoral tip at higher speeds, resulting in an exponential increase in the curvature radius.

The measurement that most closely matched the manufacturer-recommended curvature radius of 30.33 mm was 32.64 mm, achieved at a pressurization speed of 0.5 mm/s. Consequently, the optimal pressurization speed when utilizing the intraoral tip was established as 0.5 mm/s.

### 5.3. Extrusion Load at Appropriate Pressurization Speed

**Figure 14:**
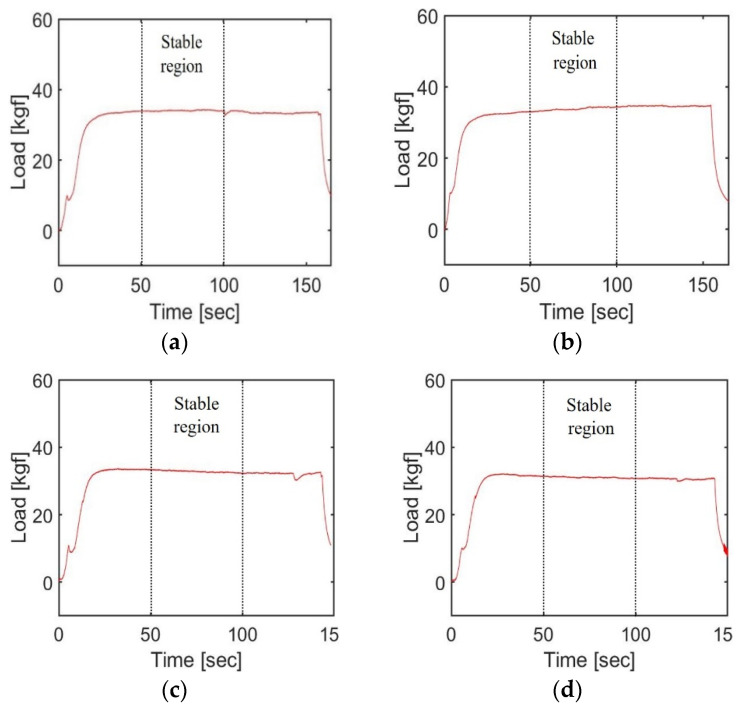
Results of total extrusion load of light type impression materials; (**a**) Mixing motor off, 1st test; (**b**) Mixing motor off, 2nd test; (**c**) Mixing motor on, 1st test; (**d**) Mixing motor on, 2nd test.

The extrusion load for light body impression material was measured at the identified appropriate pressurization speed to calculate the required torque of the main motor, specifically when the intraoral tip was used. Figure 14 illustrates the load recorded by the load cell when the cartridge was fully pressurized at a steady pressurization speed of 0.5 mm/s. Table 10 details the maximum load measured under various experimental conditions and the average load during the stable region from 50 to 100 s. The results showed that both the maximum extrusion load and the average load in the stable region were markedly higher, at 34.69 kgf and 33.78 kgf, respectively, when the mixing motor was inactive compared to its operational state.

### 5.4. Methodology for Determining the Motor Capacity

To determine the motor capacity, the following methodology was established based on previously derived equations and the results from the experiments and image processing of extrusion load and appropriate pressurization speed:

(1) Understand the power transmission mechanism of the electric dispenser and develop an equation to calculate the motor’s necessary torque and rotational speed based on the extrusion load of the impression material and pressurization speed.

(2) Utilize the load measurement system to assess the extrusion load (force applied to the cartridge) of the impression material, considering the type of material, mixing tip, operation of the mixing motor, and whether an intraoral tip is mounted.

(3) Analyze the maximum extrusion load and average load during a stable period from the measurement results.

(4) Conduct experiments to identify the optimal pressurization speed in scenarios prone to leakage and component damage. Adjust pressurization speeds through servo motor control and record videos of the extrusion trajectory of the impression material.

(5) Process the recorded footage. Capture the trajectory during the stable extrusion phases, extract pixel coordinates from RGB information, apply curve fitting to represent these coordinates as a single line, and measure the trajectory’s curvature radius in the same area using circle fit code.

(6) Determine the pressurization speed that produces a curvature radius close to the manufacturer’s recommended radius.

(7) Apply the obtained maximum extrusion load and appropriate pressurization speed to the equation from step (1) to calculate the necessary torque and suitable rotational speed of the motor.

This method was implemented, and step (7) was executed. The study’s results were applied to the previously derived Equations (4) and (7) as follows.

The maximum extrusion load obtained experimentally, 16.52 kgf without an intraoral tip and 34.69 kgf with light body impression material and an intraoral tip, was used. A safety factor of 2.5 was considered for the main motor gearbox efficiency and gear transmission losses. When calculating the final power transmission efficiency using the respective average efficiency of the worm gear, rack, and motor gearbox used in the dispenser, it was determined to be approximately 45%. Therefore, to ensure that the system operates safely under maximum load conditions, the safety factor and torque margin were, respectively, set at 2.5 and 150%. Consequently, the required torque for the main motor was calculated to be 0.89 kgf·cm without the intraoral tip, and 1.86 kgf·cm when using the intraoral tip with the light body impression material.

The suitable rpm was determined by applying the experimentally identified pressurization speed of 0.5 mm/s, and other dimensions were applied similarly. Thus, the suitable rpm for the main motor when using the light body impression material with an intraoral tip was calculated as 22.28 rpm.

Considering that the original motor had a rated torque of 6.20 kgf·cm and rotational speed of 50 rpm, it was evident that the capacity of the newly calculated motor was significantly lower. When the intraoral tip was not attached, the recalculated motor capacity required 85.64% less torque compared to the rated capacity of the original motor. With the attachment of the intraoral tip, the required torque was reduced by 70.00% and the rotational speed decreased by 55.44%.

## 6. Conclusions

This study proposes a methodology for determining drive motor capacity by integrating the analysis of extrusion behavior based on image processing with mechanical mechanism analysis for back-calculating motor specifications.

Traditional design approaches for dental dispensers lack appropriate standards based on the principles of mechanical engineering. The presented methodology offers a clear rationale based on experimental data to prevent the overdesign of dispenser drive motors. It also establishes criteria for the optimal extrusion speed based on observations of the extrusion behavior of impression materials, and suggests an intuitive method using image data for determination. This approach can be universally applied to impression materials with varying properties, yielding significant results. The results of this study reveal that significantly lower torque and reduced rotational speed are required for the extrusion of impression materials compared to the rated capacity of existing motors using this methodology.

The potential applications of this method extend beyond the optimal selection of motors for dental impression material dispensers to contribute towards the design of more compact dispensers through reduced power consumption and lightweighting of motors and batteries.

## Figures and Tables

**Table 1:** Specifications of testbed.

Parts	Specification
Servo motor	1.7A/3000 rpm/3.12 kgf·cm
Linear actuator	200 stroke/max. 40 kgf/200 mm/s
Load cell	max.100 kgf/1.0 ± 0.01 mV/V
Mixing motor	0.62A/624 rpm/0.50 kgf·cm

**Table 2:** Characteristics and physical properties of elastomeric impression materials (ISO 4823).

Type	Consistency (mm)		Detail Reproduction (μm)	Linear Dimensional Change (%)	Compatibility with Gypsum (μm)	Elastic Recovery (%)	Stain in Compression (%)	
	Min.	Max.		Max.		Min.	Min.	Max.
0	-	35	75	1.5	75	96.5	0.8	20
1	-	35	50	1.5	50	96.5	0.8	20
2	31	41	20	1.5	50	96.5	2.0	20
3	36	-	20	1.5	50	96.5	2.0	20

**Table 3:** Dental impression materials used in study.

Product	Composition	Type	Density	Manufacturer
Denta-sil Light body LV	Polyvinyl siloxane	3	$0.00118\;g/{mm}^{3}$	Sungbotech, Daegu, Republic of Korea
Denta-sil Tray Hard		2	$0.00132\;g/{mm}^{3}$	

**Table 4:** Dimensions of light body mixing tip and intraoral tip (mm).

Light Body Mixing Tip		Intraoral Tip	
A: overall height	41.52	F: vertical length of curved tube	13.03
B: width	19.01	G: horizontal length of curved tube	19.85
C: overall length	34.10	H: radius of curvature	30.33
D: diameter of inlet	2.84	I: diameter of inlet	1.15
E: diameter of outlet	3.60	J: diameter of outlet	0.40

**Table 5:** Comparison of outlet area.

Intraoral Tip	Area of Outlet [mm^2^]
w/o	10.17
w/	0.50

**Table 6:** Experimental conditions of load measurement.

Case	Impression Materials	Intraoral Tip	Mixing Motor
1	Light body LV	w/o	On
2			Off
3	Medium body—Tray Hard		On
4			Off

**Table 7:** Experimental conditions of pressurization speed.

Case	Impression Materials	Intraoral Tip	Mixing Motor	Pressurization Speed (mm/s)
1	Light body LV	w/	On	0.8
2				0.7
3				0.6
4				0.5
5				0.4
6				0.3

**Table 8:** Results of maximum extrusion load.

Case	Maximum (kgf)	Average Value of Stable Region (kgf)
1	16.05	14.21
2	14.02	12.37
3	16.52	15.84
4	14.94	13.46

**Table 9:** Results of radius of curvature.

Pressurization Speed (mm/s)	Average Value of Radius of Curvature (mm)
0.8	111.08
0.7	71.63
0.6	46.70
0.5	32.64
0.4	19.20

**Table 10:** Results of extrusion load.

Case	Maximum (kgf)	Average Value of Stable Region (kgf)
1	34.69	33.78
2	32.98	32.21

## Data Availability

The data presented in this study are available on request from the corresponding author and the first author (due to privacy).

## References

[1] Kim S.H. (2006). A study on the physical properties of addition silicone impression materials. J. Dent. Hyg. Sci..

[2] Choi T.S., Jeong Y.H., Lee Y.R., Song H.J., Park Y.J. (2009). Tensile strengths and rheological properties of commercial addition polymerization silicone rubber impression materials. Korean J. Dent. Mater..

[3] (2021). Dentistry—Elastomeric Impression and Bite Registration Materials.

[4] Lim K.C., Chong Y.H., Soh G. (1992). Effect of operator variability on void formation in impressions made with an automixed addition silicone. Aust. Dent. J..

[5] Youn M.G., Youn S.H. (2018). Dental Impression Material Electric Dispenser. KR Patent.

[6] Ryu H.S., Lim H.S., Lim J.H., Cho I.H. (2002). Study on void formation and detail reproduction according to various impression materials and mixing methods. J. Korean Acad. Prosthodont..

[7] Hamalian T.A., Nasr E., Chidiac J.J. (2011). Impression materials in fixed prosthodontics: Influence of choice on clinical procedure. J. Prosthodont. Implant Esthet. Reconst. Dent..

[8] Mandikos M.N. (1998). Polyvinyl siloxane impression materials: An update on clinical use. Aust. Dent. J..

[9] Lu H., Nguyen B., Powers J.M. (2004). Mechanical properties of 3 hydrophilic addition silicone and polyether elastomeric impression materials. J. Prosthet. Dent..

[10] Surapaneni H., Attili S. (2013). Polyvinyl siloxanes in dentistry: An overview. Trends Biomater. Artif. Organs.

[11] Craig R.G. (1985). Evaluation of an automatic mixing system for an addition silicone impression material. J. Am. Dent. Assoc..

[12] Keck S.C. (1985). Automixing: A new concept in elastomeric impression material delivery systems. J. Prosthet. Dent..

[13] Di Felice R., Scotti R., Belser U.C. (2002). Influence of the mixing technique on the content of voids in two polyether impression materials. Schweiz. Monatsschr. Zahnmed..

[14] Zelikman H., Rosner O., Naishlos S., Azem H., Meinster I., Glikman A., Matalon S. (2021). Effect of mixing and impression techniques using vinyl polysiloxane (VPS) on the accuracy of fixed partial dentures. Appl. Sci..

[15] Lepe X., Johnson G.H., Berg J.C., Aw T.C. (1998). Effect of mixing technique on surface characteristics of impression materials. J. Prosthet. Dent..

[16] Nam J., Raigrodski A.J., Townsend J., Lepe X., Mancl L.A. (2007). Assessment of preference of mixing techniques and duration of mixing and tray loading for two viscosities of vinyl polysiloxane material. J. Prosthet. Dent..

[17] Maluly-Proni A.T., Delben J.A., Briso A.L.F., Marson F.C., Dos Santos P.H. (2022). Evaluation of material waste, dimensional stability, and detail reproduction of polyvinyl siloxane impression materials mixed with different mixing tips. J. Prosthet. Dent..

[18] McCabe J.F., Arikawa H. (1998). Rheological properties of elastomeric impression materials before and during setting. J. Dent. Res..

[19] German M.J., Carrick T.E., McCabe J.F. (2008). Surface detail reproduction of elastomeric impression materials related to rheological properties. Dent. Mater..

[20] Martinez J.E., Combe E.C., Pesun I.J. (2001). Rheological properties of vinyl polysiloxane impression pastes. Dent. Mater..

[21] Kang J.K. (2001). Comparative study on properties of commercial polyvinyl siloxane impression materials. J. Korean Soc. Dent. Hyg..

[22] Kim K.M., Lee J.S., Kim K.N., Shin S.W. (2001). Dimensional changes of dental impression materials by thermal changes. J. Biomed. Mater. Res..

[23] Corso M., Abanomy A., Di Canzio J., Zurakowski D., Morgano S.M. (1998). Effect of temperature changes on the dimensional stability of polyvinyl siloxane and polyether impression materials. J. Prosthet. Dent..

[24] Jang J.S. (2016). Dynamic Analysis and Experimental Investigation of Cables with Time-Varying Unwinding Velocity. Doctoral Thesis.

[25] Kiusalaas J. (2015). Numerical Methods in Engineering with MATLAB^®^.

[26] Banaszek A., Petrović R. (2010). Calculations of the Unloading Operation in Liquid Cargo Service with High Density on Modern Product and Chemical Tankers Equipped with Hydraulic Submerged Cargo Pumps. J. Mech. Eng./Strojniški Vestnik..

[27] Banaszek A., Urbanski T. (2020). The flow calculation algorithm of submerged hydraulic cargo pumps working with reduced pump speed on modern product and chemical tankers. Procedia Comput. Sci..

[28] Jin M.H., Lee K.R. (2014). Comparison of shear rate and viscosity of commercial dental impression materials. J. Ind. Technol. Kangwon Natl. Univ. Kore..

[29] Kang C.W., Yang K.S. (2013). Numerical study of laminar flow and heat transfer in curved pipe flow. Trans. Korean Soc. Mech. Eng.-B.

[30] Sohn H.C., Lee H.N., Park G.M. (2005). Axial velocity profiles and secondary flows of developing laminar flows in a straight connected exit region of a 180° square curved duct. Trans. Korean Soc. Mech. Eng.-B.

